# Generating and validating renewable affimer protein binding reagents targeting SH2 domains

**DOI:** 10.1038/s41598-024-79357-4

**Published:** 2024-11-16

**Authors:** Sophie J. Heseltine, Gregory J. Billenness, Heather L Martin, Christian Tiede, Anna A.S. Tang, Eleanor Foy, Grace Reddy, Naomi Gibson, Matt Johnson, Michael E. Webb, Michael J. McPherson, Darren C. Tomlinson

**Affiliations:** 1 School of Molecular and Cellular Biology, University of Leeds, Leeds, UK; 2 Astbury Centre for Structural and Molecular Biology, University of Leeds, Leeds, UK; 3 School of Chemistry, University of Leeds, Leeds, UK; 4 Avacta Life Sciences, Wetherby, UK

**Keywords:** Biological techniques, High-throughput screening, Imaging, Cell signalling, Cellular imaging

## Abstract

Despite SH2 domains, being pivotal in protein interactions linked to various diseases like cancer, we lack specific research tools for intracellular assays. Understanding SH2-mediated interactions and creating effective inhibitors requires tools which target individual protein domains. Affimer reagents exhibit promise, yet their potential against the extensive SH2 domain family remains largely unexplored. Our study aimed to bridge this gap by identifying Affimer reagents that selectively bind to 22 out of 41 SH2 domains. These reagents enabled a medium-throughput screening approach resembling siRNA studies, shedding light on their functionality. Notably, select Affimers demonstrated the ability to curtail the nuclear translocation of pERK, with Grb2 being a prominent target. Further analyses revealed that these Grb2-specific Affimer reagents displayed competitive inhibition with impressive metrics: IC50s ranging from 270.9 nM to 1.22 µM, together with low nanomolar binding affinities. Moreover, they exhibited the ability to pull down endogenous Grb2 from cell lysates, illustrating their efficacy in binding the Grb2 SH2 domain. This comprehensive assessment underscores the potential of Affimer reagents as domain-specific inhibitors. Their viability for medium/high-throughput phenotypic screening presents a promising avenue via which to identify and characterize potential drug targets within the SH2 domain family.

**Supplementary Information:**

The online version contains supplementary material available at 10.1038/s41598-024-79357-4.

## Introduction

Src Homology 2 (SH2) domains are phosphotyrosine (pTyr)-binding modules found in over 120 human proteins^1^. Approximately 100 amino acids in length, the SH2 structure consists of a central anti-parallel β-sheet flanked on both sides by an α-helix^2^. These form two binding sites; a conserved pTyr binding pocket and a variable pocket that binds residues C-terminal to the pTyr. In total, a four to seven amino acid motif is bound by the domain^3^. SH2s are usually found in conjunction with either catalytic domains or other binding domains, such as SH3s^4^. SH2 domains constitute the largest class of pTyr-binding modules and are found in a wide variety of proteins including kinases, adaptor proteins, transcription factors and phosphatases^5,6^. Through their binding of phosphorylated targets, they mediate protein-protein interactions (PPIs) and are involved in numerous intracellular signalling pathways. Many of these SH2-regulated interactions play key roles in processes that become dysregulated in disease, such as cell cycling, proliferation and apoptosis^7,8^. SH2 domains are therefore potential therapeutic targets for the treatment of several disorders including cancer, and study of their function could lead to a better understanding of numerous cancer signalling pathways^9,10^.

Though recognised as promising disease targets, there is still a lack of research reagents available for SH2 domains^11^ and the scarcity of SH2 inhibitors that are effective in intracellular assays has hampered study of SH2-mediated mechanisms^2,12^. The highly conserved sequence and structure of SH2 domains raises significant challenges in generating specific binding reagents or inhibitors^2,13^. As a result, the function of many SH2s has not yet been explored^14^ and it is widely acknowledged that protein-specific SH2 inhibitors would be highly valuable research tools that would enable the discovery of novel biology and new pharmaceutical targets in various disorders^1,15^. The development of these could also lead to the detection of residues that may be exploitable for protein-specific drug design^11^.

Analysis of intracellular protein function can be achieved through techniques such as gene knockout or RNA interference. However, these methods are impractical for studying domain-specific interactions as they result in the deletion of the entire protein^12^. In order to observe the cellular functions of SH2 domains, binding molecules acting at the protein level are needed^14^. In recent years, the development of scaffold based binding proteins (SBPs) has aided targeted disruption of PPIs^15–17^. SBPs have many advantageous features; ability to be expressed intracellularly, high solubility, high stability and lack of disulphide bonds, and include, amongst others, Designed Ankyrin Repeat Proteins (DARPins) and monobodies as well as the Affimers^18–20^ utilised in this study. These easily producible proteins have been used for a range of biological and therapeutic applications to date^21^. Monobodies targeting the SH2 domain of Abl have been shown to bind Bcr-Abl allosterically and inhibit its function resulting in apoptosis of chronic myeloid leukaemia cells^22,23^. Our group have previously identified Affimers that bind the SH2 domains of the Grb family of proteins and the individual SH2 domains of PI3K^19^. These studies demonstrate the potential for SBPs in modulation of SH2 mediated signalling events. However, to date, there has not been available a toolbox of SH2 modulating reagents to allow the roles of individual SH2 containing proteins, and even individual domains, to be delineated in desired cellular phenotypes.

Here we have developed a toolbox of Affimer reagents that bind 38 SH2 domains representing approximately a third of the known SH2-domain containing proteins. The specificity of the toolbox is comparable to those seen with ScFv screens^24^ and we have proved that the toolbox functions intracellularly by the identification of Grb2 as a major SH2 protein in the MAPK pathway using a phenotypic screen looking at phosphorylation and subsequent nuclear translocation of ERK. These specific Grb2-binding Affimers were shown to have nanomolar affinities and IC_50_ values demonstrating the utility of the toolbox.

## Results

### High-throughput screen of SH2 domains to identify Affimer binders

Building on our previously published work identifying Affimers (see Supplementary Fig. S1 for Affimer crystal structure) that specifically bound the SH2 domains of the Grb family of proteins, p85 and p55^19^ we sought to generate a toolbox of reagents to dissect the roles of SH2 domain/proteins intracellularly and test the application of such reagents in a medium throughput screen. To achieve this an additional 32 SH2 domains encompassing the main molecular functions of SH2 domain containing proteins (excluding those associated with RAS regulation and ubiquitination)^25^ and were readily available as a set for bacterial expression (Pawson Lab (Samuel Lunenfeld Research Institute, Canada)) were selected. These 32 SH2 domains were then expressed and purified in a small-scale high throughput manner utilising 3mL cultures and the KingFisher Flex yielding between 16 and 173 µg of protein. These proteins were then used for isolation of Affimers from our phage library using a competitive approach^18^ for some targets a second non-competitive screen was also carried out. Three panning rounds were sufficient to yield substantial amplification for 18 of the targets whilst a fourth panning round increased this to 27 targets giving an 84% success rate at this stage. No amplification was achieved for Ptpn11-N, Src2, Syk-C, Stat2 and Yes, possibly owing to poor protein quality/quantity or low levels of biotinylation. Target binding for 24–48 clones for the 27 successful targets was assessed by phage ELISA (see Supplementary Fig. S2) and successful binders sent for sequencing to identify unique clones, yielding 621 unique clones. The range of clones per target varied from 1 to 48 (Table 1).

**Table 1:** Summary of phage display screening and isolation of Affimer clones to BAP-tagged SH2 domains.

SH2 target	Phage ELISA hit rate (%)	Number sequenced	Unique clones
Abl1	60	48	6
Abl2	100	48	42
Bmx	98	48	22
Crk	83	48	21
Fyn	90	48	20
Grb2	92	48	30
Grb7	73 / 15	61	48
Grb10	42 / 13	34	12
Grb14	92 / 10	52	8
Lck	91	87	35
Lyn	8	8	4
Nck1	81	48	4
Nck2	15	8	7
p85α-C	23	48	9
p85α-N	92	48	24
p85β-C	54	48	8
p85β-N	98	48	8
p55γ-C	81	48	13
p55γ-N	100	48	35
PLCγ1-T	27 / 4	21	13
PLCγ1-N	88	10	2
PLCγ2-T	8	6	3
PLCγ2-N	48 / 4	33	11
She	66	48	8
Ship1	52 / 50	54	26
Ship2	25 / 25	36	27
Src1	17	8	4
*Stat1*	*2*	*48*	*14*
Stat3	92	48	47
Stat4	31	48	48
*Stat5a*	*29*	*7*	*2*
Stat5b	2	6	5
*Stat6*	*4*	*1*	*1*
Syk-N	69	42	18
Tec	4	7	6
*Tns1*	*38*	*48*	*4*
Vav1	81	48	26

Final hit rates and unique clones isolated for each successfully screened SH2 domain. Targets for which positive hit criteria was lowered to ≥ 3x that of the negative control (compared to the standard criteria of ≥ 10x that of the negative control) are highlighted in italics. For SH2 domains screened twice, competitively and non-competitively, both hit rates are shown in column 2.

**Fig. 1:**
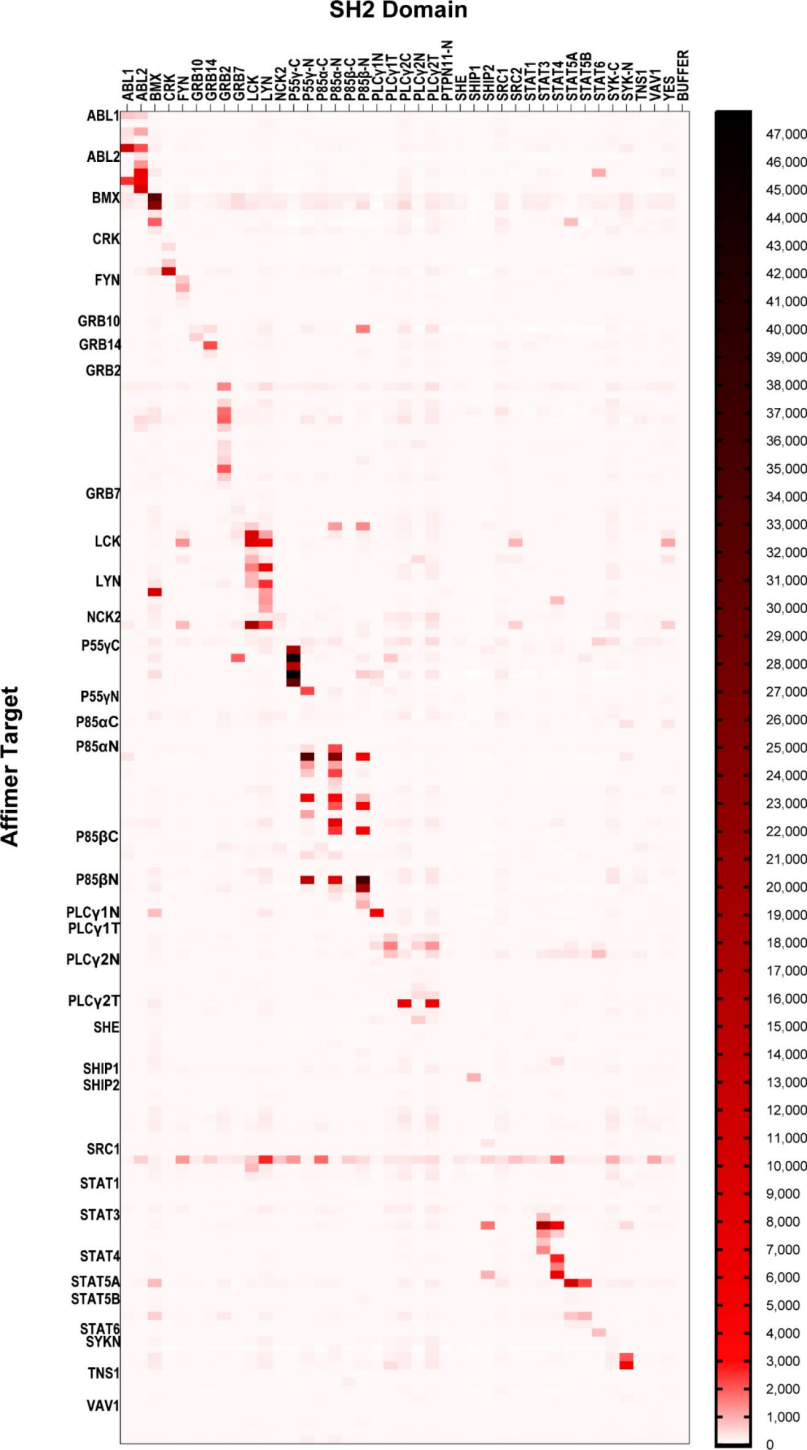
Specificities of SH2-binding Affimers tested against 40 SH2 domains. Heatmap of the results of SH2 protein microarrays testing the specificity of SH2-binding Affimers. The heatmap displays the mean background corrected fluorescent signal at 635 nm for each Affimer against each SH2 domain, normalised to the buffer only control, as shown in the scale bar. *n* = 3 independent experiments.

As SH2 domains share high structural homology^25^, a microarray approach was used to determine the specificity of these binders for their target domain in a rapid fashion. BAP-tagged SH2 domains were printed onto streptavidin coated slides, five spots for each SH2 domain, 10 buffer spots per array and 14 arrays per slide. Three SH2 domains, Nck1, Stat2 and Tec, were excluded from the microarray as the detection antibody bound to these proteins in optimisation experiments. The six SH2 domains we had previously targeted^19^ were also included on the microarrays, giving a total of 35 SH2 domains. A maximum of five clones per screen per SH2 domain were then tested for specificity (clones were selected based on their sequence and signal in the phage ELISA), with the exception of the Grb2-binding and p85N-binding Affimers identified in our previous work^19^ that were all tested. HA-tagged Affimers (5 µg/ml) were applied to microarrays and detected with an anti-HA antibody (1 µg/ml). Of the 162 Affimers tested, 54 showed no binding at the cutoff of 50x the signal from buffer only spots (Fig. 1).

This was in contrast to the phage ELISAs used in Affimer identification, however this appeared to be target dependent with four targets that showed no binding (She, Tns1, p85α-C and p85β-C) that was possibly the result of protein denaturation^26–28^. Re-testing these non-binding Affimers in an ELISA format only identified 14 that bound their targets, however these included all Affimers targeting p85α-C suggesting this target had indeed become denatured on the microarray. From the remaining 108 Affimers that showed binding in the microarray, 51 showed specificity for their respective target (Fig. 1). Affimer reagents were deemed specific if off-target interactions were ≤ 10% of the signal shown for the intended target, in accordance with previous work on SH2 domain-binding antibody fragments^11,24^. In total, specific Affimers were found for 22 SH2 domains giving a specific hit rate of 63% (Table 2).

**Table 2:** Target-specific Affimer clones as determined by SH2 protein microarray.

Target	Number of specific clones
Abl1	1
Abl2	5
Bmx	4
Crk	3
Fyn	4
Grb2	5
Grb10	1
Grb14	2
Lyn	3
P85α-N	2
P85β-N	1
P55γ-C	5
P55γ-N	1
PLCγ1-N	1
PLCγ2-T	1
PLCγ2-N	1
Ship1	1
Ship2	1
Stat3	3
Stat4	3
Stat6	1
Syk-N	2

Table summarising specific Affimer clones for SH2 targets as identified by protein microarray. Clones were deemed specific if off-target interactions showed a signal < 10% of that of the intended target. Also see Supplementary Table S1 for variable region sequences.

### Intracellular pathway screening

**Fig. 2:**
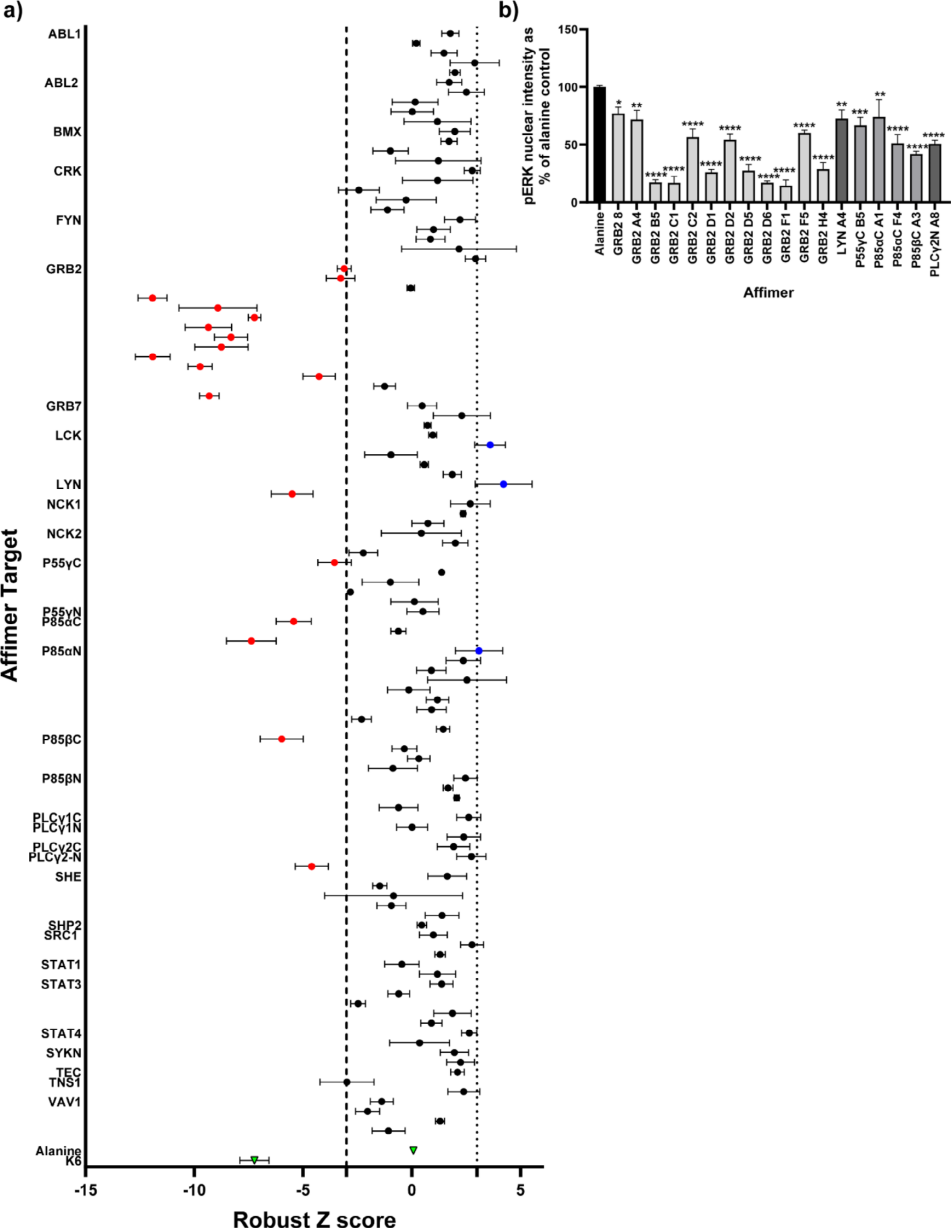
Screening of SH2-binding Affimers in the pERK translocation assay. (**a**) HEK293 cells reverse-transfected with either SH2-binding Affimers or control Affimers (green triangles; Alanine Affimer (non-binding variable regions of AAAA and AAE)- negative control; K6 - positive control) plasmids were starved and stimulated with EGF for 5 min. After staining and acquisition, images were analysed for pERK nuclear intensity in GFP positive cells about the fluorescence threshold of 400. To compare ERK phosphorylation levels between all wells on each plate the robust z-score was used, calculated as the (pERK signal of the individual well - median pERK signal of the whole plate)/ median absolute deviation of the pERK signal of the whole plate. A well returning a mean absolute robust z-score of |> 3| was considered a hit. Cells highlighted in red reduced pERK nuclear translocation whilst those highlighted in blue increased pERK nuclear translocation. (**b**) SH2-binding Affimers that reduced pERK nuclear intensity were validated by repeated experiments and the pERK signal normalised to the Alanine Affimer control. Data is expressed as mean ± SEM, *n* = 3 independent experiments (biological replicates). One-Way ANOVA with Dunnett’s post-hoc test compared to Alanine Affimer *p < 0.005, **p < 0.01, *** *p* < 0.001, ****p < 0.0001.

Having identified a toolbox of SH2-binding Affimers with good target specificity, we investigated their application towards understanding the roles of SH2 domain containing proteins in cell signalling. For this we used an assay from our previous work on the modulation of Ras with Affimers^29^, and examined the nuclear translocation of phosphorylated extracellular signal-regulated kinase (pERK) with high content imaging as a measure of EGFR signalling. By testing our toolbox of SH2-binding Affimers with this assay we hoped to identify those SH2 domain containing proteins with roles in this pathway as a proof of principle for the utility of the toolbox, in particular we anticipated that we should be able to identify Grb2 owing to its well characterised role with this pathway^30–32^. To achieve this 119 of the 162 Affimers used in the microarray were subcloned into the mammalian expression vector pCMV6-tGFP. Our previous pERK nuclear translocation assay was adapted to a screening format of four 96 well-plates with each plate featuring a maximum of 30 Affimers to 62 negative controls (a non-targeting Affimer – Alanine) and four positive controls of a Ras-inhibiting Affimer K6. HEK293 cells were reverse-transfected with these constructs and pERK nuclear translocation assessed 48 hr later. The assay quality of the screen was assessed by robust Z’ factor analysis which yielded a value of 0.52 indicating the screen had a difference of 12 standard deviations between the positive and negative controls showing that it was likely to pick up a number of hits. The screen was repeated in triplicate and 18 hits identified as those Affimers with robust Z scores of less than − 3, identifying SH2 domains involved in positive EGFR signalling (Fig. 2a). Intriguingly 3 Affimers (Lck A7, Lyn A2, p85αN C6) increased pERK nuclear translocation as indicated by robust Z scores of greater than + 3. The 18 hits were then validated by individual assessment of pERK translocation and all were confirmed as hits (Fig. 2b) including 12 Affimers targeting Grb2. It was unsurprising that Grb2-binding Affimers were the major hits from this screen as this is the predominant SH2 domain containing protein in the EGFR signalling pathway^30–32^, but this confirms the utility of this screening technique as it highlighted a known major player in this pathway. PI3K hits (p85αC A1 and F4, p85βN A3 and p55ɣC B5) were also seen and this confirms our previous work^19^ as there is a degree of cross talk between the Akt pathway activated by PI3K upon EGF stimulation and the ERK pathway^33^. The two remaining hits Plcy2N A8 and Lyn A4 have all been linked to MAPK pathway signalling in other cell types with other stimulating agents, the majority of which show heterogeneity with the EGF receptor (ErbB1)^34–36^. These signalling events are not the major pathway for ERK phosphorylation and this is reflected in the relatively small reductions in pERK compared to those seen with Grb2 (Fig. 2a). Thus, we have demonstrated the utility of using binding proteins in a high-throughput screen and that the SH2 Affimer toolbox functions in vitro to identify SH2 domains with both major and minor roles in MAPK signalling.

### Characterisation of Grb2 Affimers

**Fig. 3:**
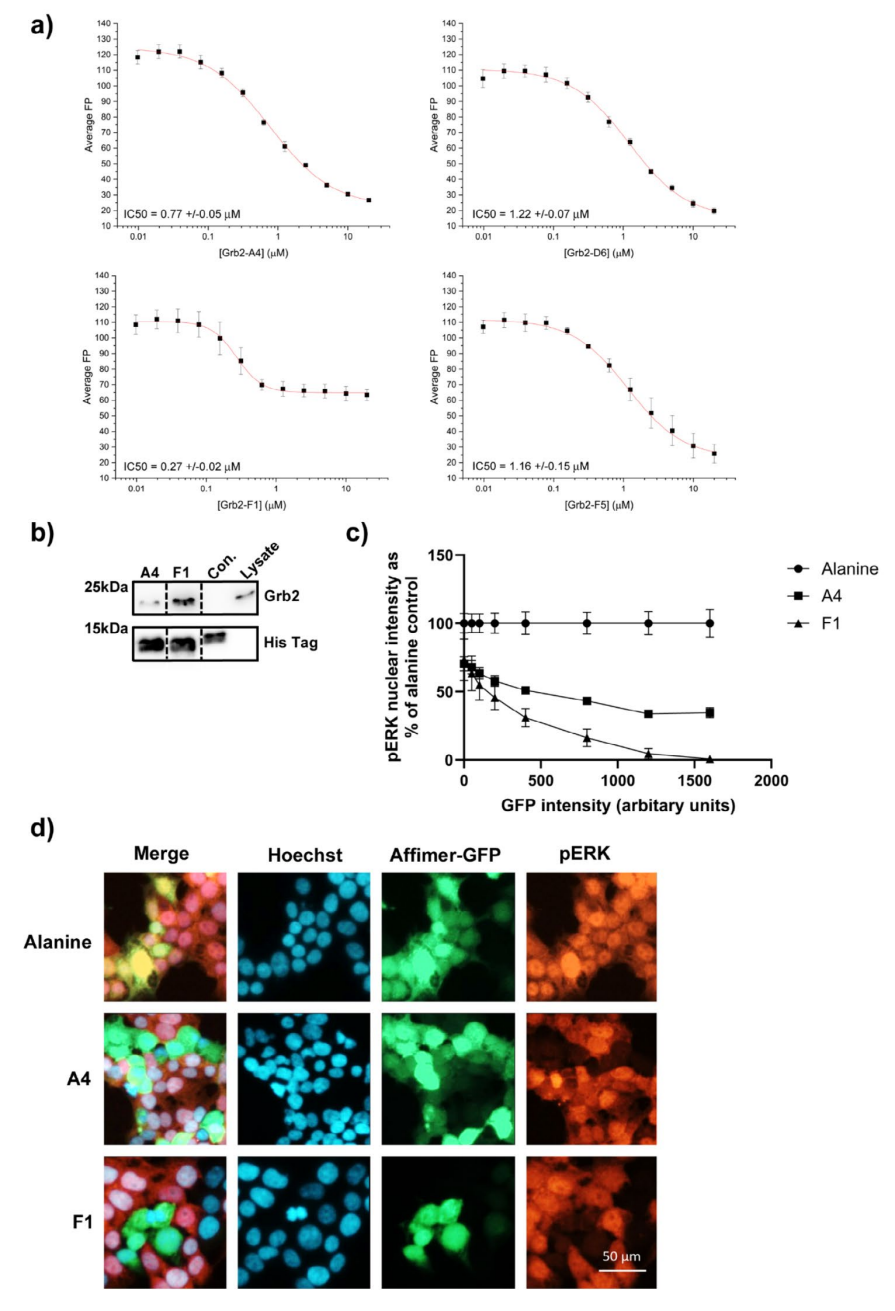
Grb2 SH2-binding Affimers show competitive inhibition of the Grb2 SH2 and immunoprecipitation of endogenous Grb2 from cell lysate. (**a**) Fluorescence polarisation (FP) was used as a measure of binding between the Grb2 SH2 and a FITC-labelled phosphopeptide ligand (FYp). A Grb2 SH2-FYp control binding curve was also read on each plate ([FYp] = 20nM) to ensure Grb2 SH2 and the FYp probe were binding as expected (see Supplementary Figure S3). Serial dilutions of Affimer were set up in triplicate and the FP measured in each well ([FYp] = 20nM; [Grb2 SH2] = 0.25µM). (**b**) Immunoprecipitation of endogenous Grb2 from HEK293 cell lysates by Grb2 SH2- binding Affimers A4 and F1. (dotted lines indicate the removal of non-relevant lanes from membrane, see Supplementary Figure S5 for full membranes) (**c**) Correlation of GFP intensity versus pERK nuclear intensity demonstrating the dose-dependent effects of Grb2 SH2- binding Affimers A4 and F1. (**d**) Representative images of Grb2 SH2- binding Affimers A4 and F1 in the pERK nuclear translocation assay are shown. Con. – Control Affimer (raised against yeast sumo). Data shown is mean ± SEM. *n* = 3 independent experiments, IC_50_s calculated using OriginPro 9.1. b), c) and d) are biological replicates *n* = 3.

Having identified Grb2 Affimers as the major hit from the pERK screen the four Affimers (variable region sequences shown in Supplementary Table S1) that had shown specificity for Grb2 (as measured by microarray) were then characterised further. Initially their competitive inhibitory capabilities were quantified by fluorescence polarisation, as measured by the displacement of a fluorescently labelled SH2 phosphopeptide from the Grb2 SH2 domain, giving IC_50_ values ranging from 270 nM (Affimer Grb2 F1) to 1.22 µM (Affimer Grb2 D6) (Fig. 3a). Affimer Grb2 F1 had the lowest IC_50_ value despite the lack of complete inhibition as evidenced by the bottom plateau not trending towards zero. The cause of this reduced inhibition requires further investigation, but it is possible that this Affimer is binding to the fluorescently labelled SH2 phosphopeptide. The two Affimers (A4 and F1) showing nanomolar IC_50_ values were taken forward for more detailed characterisation.

**Fig. 4:**
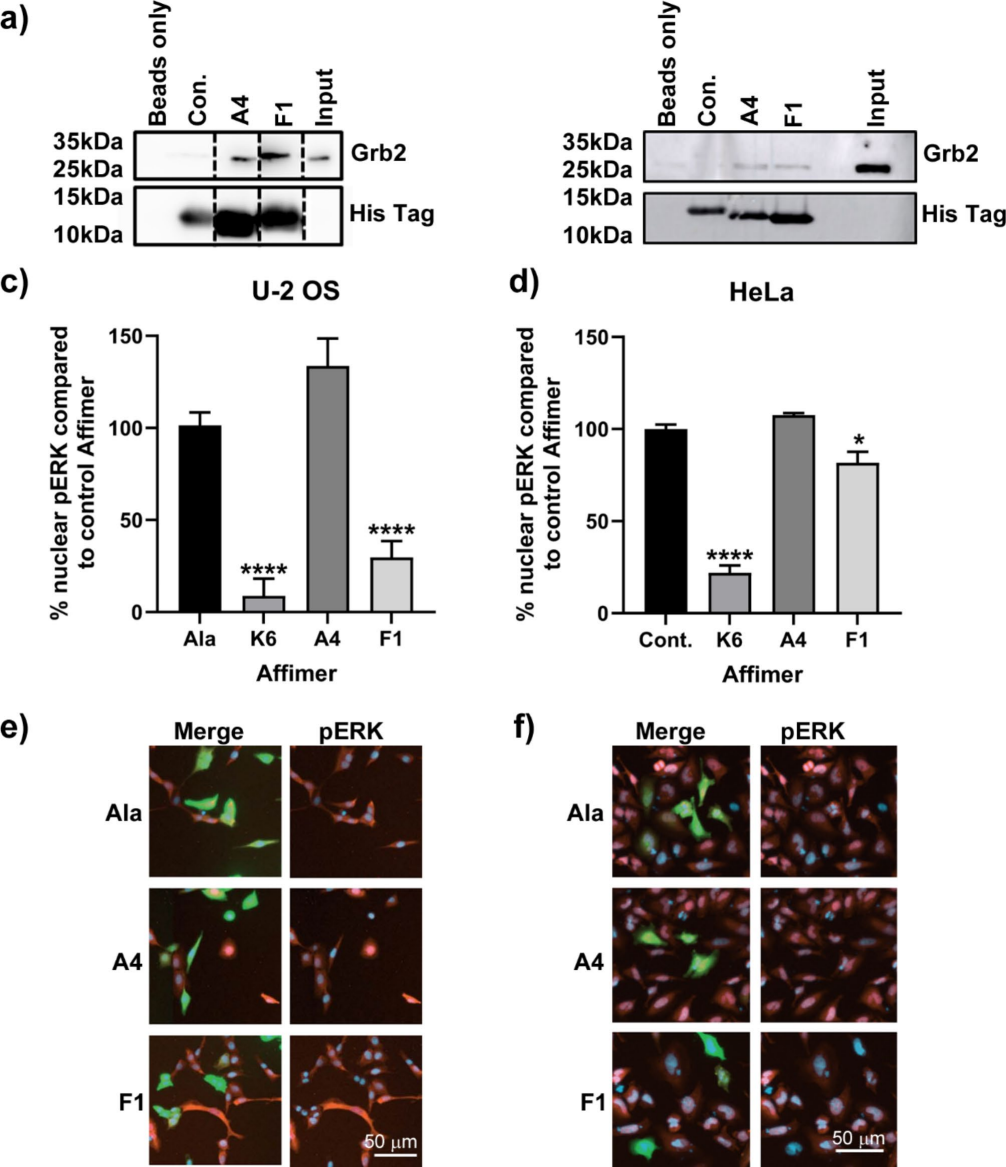
Grb2 SH2-binding Affimers immunoprecipitate endogenous Grb2 from cancer cell lysates and inhibit pERK nuclear translocation. Immunoprecipitation of endogenous Grb2 from U-2 OS (**a)**) and HeLa (**b)**) cell lysates by Grb2 SH2-binding Affimers A4 and F1. (dotted lines indicate the removal of non-relevant lanes from membrane, see Supplementary Figure S5 for full membranes) Grb2 SH2- binding Affimer F1 reduces pERK nuclear translocation when compared to the non-targeting alanine Affimer in both U-2 OS (**c**) and HeLa (**d**) cells. Representative images of Grb2 SH2- binding Affimers A4 and F1 in the pERK nuclear translocation assay are shown in (**e**) and (**f**). Green – Grb-2 binding Affimers, Red – pERK, Blue – Hoechst. Con. – Control Affimer, Ala – Alanine Affimer. Data shown is mean ± SEM. *n* = 3 independent experiments (biological replicates).

Surface plasmon resonance showed the affinity of these Affimers to full length Grb2 to be low nanomolar (A4 K_D_ = 11.8 ± 6.9 nM; F1 K_D_ = 34.8 ± 16.9 nM; see Supplementary Figure S4) comparable with the affinity of Grb2 for its intracellular targets^37^, demonstrating these Affimers are able to compete in vitro for binding of the SH2 domain of Grb2. The binding affinities seen in the SPR experiments are non-competitive and this likely explains the higher affinities recorded compared with those seen in our FP assay (Fig. 3a) where the fluorescent probe provides competition for the SH2 domain binding. In vitro binding was confirmed by the ability of these Affimers to immunoprecipitate Grb2 from HEK293 lysates (Fig. 3b and Supplementary Figure S5). Next, we explored if these Affimers demonstrated a dose-response in terms of inhibition of pERK nuclear translocation by correlating GFP intensity with pERK nuclear intensity, as GFP intensity increased, i.e. dose of Affimer, pERK nuclear intensity decreased and for both A4 and F1 the correlation was significant (Pearson correlation *p* = 0.0003 A4, *p* = 0.0004 F1; Fig. 3c and d). This demonstrates that the SH2 toolbox contains high affinity, specific Affimer binders that can block SH2 function in normal cells in a dose-dependent manner. To determine the wider relevance of these results we determined if they were applicable to cancer cells. The effects of these two Grb2 Affimers were explored in two cancer cell lines, U-2-OS and HeLa. Both A4 and F1 were able to immunoprecipitate Grb2 from both HeLa and U-2-OS lysates (Fig. 4a and b, and Supplementary Fig. S5). However, only F1 was able to inhibit pERK nuclear translocation in both cell lines (One-way ANOVA with Dunnett’s post hoc test *p* < 0.0001 for U-2 OS and *p* = 0.0237 for HeLa; Fig. 4c–f). These data are consistent with A4 being a marginal hit in the screen (z = − 3.27 ± 0.66). The degree of inhibition of pERK nuclear translocation achieved by Affimer F1 varied between the cell lines tested, with similar levels of inhibition achieved in HEK293 and U-2 OS cells (84% and 78% respectively), but only 18% in HeLa cells. The reason behind these differences is unclear and will require further investigation, but may arise of differential EGFR expression. EGFR expression is increased in HeLa cells compared to HEK293 and U-2 OS cells^38^ and this may result in increased signal transduction above the levels that can be blocked by expressed level of Grb2-binding Affimer F1. Alternatively, there may be alterations to the protein processing of the Affimer in these cells, potentially a lack of phosphorylation of the tyrosine residue in variable region 1, affecting the Affimer’s ability to bind the Grb2 SH2 domain.

## Discussion

Isolating specific and potent SH2 inhibitors has proven a significant challenge in the past, to the extent that SH2 domains were deemed ‘undruggable’ targets^9,39^. However, the development of scaffold based binding reagents, SBPs, has allowed the specific targeting of interaction domains previously abandoned as disease targets. In this work, we have created a toolbox of SH2-binding Affimers to aid in the exploration of the roles of SH2 domain-containing proteins in cellular signalling and function and to determine the ability to use this type of reagents set in high-throughput phenotypic screens. To achieve this, we successfully isolated protein-specific Affimer binders to 22 SH2 domains. This success was not only due to the stringent phage display process used, but also the incorporation of an N-terminal BAP tag on the SH2 antigens. This allowed site-directed in vivo biotinylation of the target protein for phage display screening, thus removing the need for chemical biotinylation, which has previously resulted in the coupling of a biotin molecule to free lysines in the SH2 domain binding site. This method also allows the presentation of the target protein in its native conformation, an advantage when isolating Affimers that will function in cell-based assays. The hit rate of 80% achieved is higher than previously reported hit rates from SH2 domain screening^24^. The specific hit rate of 63% is comparable to previous screens using ScFvs^5,24^ and both these studies only targeted 20 SH2 domains. An Abl SH2-binding monobody isolated by Wojcik et al.^23^ showed cross-reactivity to three other SH2s in a protein microarray used at a tenth of the concentration of the Affimers in this study, indicating that the specificity of SH2-binding Affimers is favourable when compared with similar non-antibody reagents raised against SH2s. Additionally, this monobody could not distinguish between Abl1 and Abl2 unlike some of the Abl binding Affimers identified in this study.

Whilst the previous screens identified SH2 binders, no assessment of their function in vitro in live cells was undertaken as these screens identified ScFvs that bound SH2 domains in lysates or fixed cells^5,24^. This was important for the utility of the SH2 binding Affimer toolbox. Shp2 SH2 binding monobodies have been shown to be functional in inhibiting ERK phosphorylation in HCC1171 lung cancer cells^17^ and our previous work^19,29^ show SBPs function intracellularly. This intracellular functionality was utilised to screen the SH2 Affimer toolbox in a pERK translocation assay. Popular methods for investigating MAPK signalling include western blotting and SRE luciferase assays, which can be slow and labour intensive^40^ so high-content imaging was used together with GFP-tagged SH2-binding Affimer constructs yielding a simple, time efficient and sensitive assay. This approach is easily modified to screen different endpoints, for example modulation of the phosphatidylinositol 3-kinase (PI3K)/AKT pathway by using AKT phosphorylation as the measurable endpoint^40^, or any other phenotypic change that can be imaged or measured. The identification of 18 Affimers for 6 SH2 domains that reduced EGF-induced pERK translocation indicating they inhibited MAPK signalling demonstrates a hit rate of 22%. These included 12 Affimers isolated against the Grb2 SH2 domain which is not surprising given the canonical role of Grb2 in Ras-activated MAPK signalling^30–32^. Of the remaining six Affimers four were isolated against the C-terminal SH2 domains of PI3K subunits p55ɣ (B5), p85α (A1 and F4) and p85β (A3) in conjunction with our previous work showing Affimers binding the N-terminal SH2 of p85 increase AKT phosphorylation (pAKT) levels^19^. Stimulation of the PI3K pathway leads to phosphorylation and activation of its downstream effector AKT. Activated AKT has been shown to inhibit ERK phosphorylation via its interaction with RAF^33^. Both the N and C-terminal subunits are involved in inhibition of the catalytic p110 subunit^42,43^. This suggests a mechanism where Affimer binding of the PI3K regulatory subunit’s SH2 domain leads to increased AKT activity and subsequent inhibition of ERK phosphorylation.

Characterisation of the specific Grb2 Affimers identified as inhibiting pERK translocation showed IC_50_ values ranging from 0.27 to 1.22 µM, as measured by fluorescence polarisation, which is in line with the IC_50_ values for Grb2 SH2-binding phosphopeptides^44^. The most potent Affimer, F1, had an IC_50_ comparable to bicyclic peptide inhibitors of the Grb2 SH2 domain^45^. This demonstrates that the inhibitive ability of the Grb2 SH2 Affimers is equal to, or surpasses, previously developed Grb2 SH2 inhibitors. The nanomolar affinities of the Grb2 Affimers for Grb2 is in line with that of the antibody fragments that bind the Grb2 SH2 with K_D_s in the nanomolar range^24^, the SHP2 SH2 domain binding monobodies^17^, as well as phosphopeptide binders^44^. Higher picomolar affinities have been achieved with Grb2 SH2 small molecule inhibitors that mimic the phosphorylated tyrosine residue in Grb2 SH2 binding partners^46^. In spite of their high affinity for Grb2 SH2, the small scFv antibody fragments lacked the ability to immunoprecipitate endogenous Grb2 from clarified lysate, in contrast to the Grb2-SH2 Affimers tested in this study that were able to pull out detectable levels of endogenous Grb2 from cell lysates from multiple cell lines. These results show the ability of the Affimers to bind low levels of the target, in the context of the whole protein rather than just the isolated SH2 domain. This has positive implications for their use in functional cell-based assays as we have shown with successful inhibition of EGF-stimulated MAPK signalling as measured by pERK translocation.

The Grb2 SH2 domain binds its natural substrates via selective recognition of the binding motif pY-X-N-X^10^, a motif that is mimicked by the phosphopeptide binders^44^ and the small molecule inhibitors^46^. The majority of the Grb2 SH2 Affimers share this binding motif (10/16) including Affimer F1. Interestingly Affimer A4 contains an alternative aromatic residue, tryptophan, and Affimer F5 does not contain this sequence, so this motif alone does not confer Affimer specificity for the Grb2 SH2 domain. Relating results to the specificity motifs in the variable regions of the strongest hit, Affimer F1, reveals the sequence of Y-V-N-V, as in previous work using phosphorylated peptide libraries the sequence pY-V-N-V to have the highest affinity for the Grb2 SH2^47,48^. The level of MAPK inhibition seen in this study was closely correlated with similarity to this sequence. The variable regions of A6 and H1, which failed to significantly reduce pERK, show little similarity to this sequence. This provides strong evidence that the effects seen in this assay are due to the binding of the Grb2 SH2 domain, rather than some unknown off-target effects. Importantly, Affimer reagents can utilize this motif to bind the Grb2 SH2 domain with high specificity without the need for the highly polar phosphorylated tyrosine residue, which can cause promiscuous binding^47–49^. It is possible that this motif is phosphorylated during Affimer production/expression rendering Affimer F1 even more like the natural substrate for Grb2.

In conclusion this study has demonstrated that Affimers can be isolated that bind SH2 domains in a protein-specific manner with high affinity. The specificity of Affimers for their target SH2 over highly homologous SH2 domains of other proteins and their ability to bind endogenous Grb2 is favourable when compared with previously isolated binding reagents^23,50^. Furthermore, Grb2 SH2-binding Affimers show the ability to inhibit target function. This, in conjunction with the ability of Affimers to fold correctly and bind targets in the cytoplasm, indicates that the SH2-targeting Affimer toolbox or an Affimer toolbox to other protein domains, will be useful for functional cell-based assays to determine the role of different protein domains in biology and disease. This may show the way for future development of proteome domain screening tools for functionally dissecting pathways and identifying key domains on proteins for targeted therapeutics.

## Methods

### SH2 domain production

SH2 domains were produced as previously described^19^. Briefly sequences encoded in kanamycin-resistant pET28 SacBAPvectors were purchased from the Pawson Lab (Samuel Lunenfeld Research Institute, Canada) and a biotin acceptor peptide (BAP) sequence was cloned into the vectors to give an N-terminal BAP-Histag-SH2 domain sequence. For production in Rosetta™2 (DE3) cells (Novagen, Merk Millipore), overnight starter cultures were grown at 37 °C, 230 rpm in TB medium supplemented with kanamycin (50 µg/ml), chloramphenicol (34 µg/ml), and 1% glucose. These were used to inoculate 3 ml cultures of TB kanamycin that were grown at 37 °C, 230 rpm until OD600 reached ca. 1.5 and temperature was reduced to 18 °C for 1 h before addition of 0.5 mM IPTG and cultures were grown overnight at 18 °C, 230 rpm. His-tagged SH2 proteins were purified from clarified culture lysates on a KingFisher™ Flex robotic platform (ThermoFisher) using His Mag Sepharose Ni beads (GE Healthcare), washed (50 mM NaH_2_PO4; 500 mM NaCl; 20 mM imidazole; pH 7.4) and eluted in 130 µl elution buffer (50 mM NaH_2_PO4; 500 mM NaCl; 300 mM imidazole; 10% glycerol; pH 7.4). The elution buffer also contained 1 mM TCEP. Samples were flash frozen in liquid nitrogen and stored in aliquots at − 80 °C.

### Phage display and phage ELISA

Phage display was completed over four panning rounds, as described previously^19^. Streptavidin-coated wells were used for the first panning round, followed by Streptavidin-coated magnetic beads (Dynabeads®; Life Technologies) and NeutrAvidin-coated wells in the final panning round. For competitive pans, an additional incubation of target-bound phage with 2.5 µg of non-biotinylated target was performed for 24 h at room temp before elution. Phage ELISAs were conducted as described previously^19^, and positive clones sent for sequencing.

### Affimer production

Affimer sequences were cloned into pET11a using the NheI and NotI sites. SH2-binding Affimers were produced in BL21 STAR™ (DE3) E. coli (C601003, Life Technologies, Invitrogen) and affinity purified using Ni-NTA resin as previously described^19^. For HA-tagged Affimers, Affimer sequences were subcloned into kanamycin-resistant pET-lectra vectors with C-terminal HA, 8xHis-tag sequences and produced in BL21 Star™ (DE3) E.coli cells in 24 well plates. Briefly, 200 µl starter cultures were grown at 37 °C, 1050 rpm in a 96-well plate for 6–8 h in LB broth kanamycin (50 µg/ml) + 1% glucose. Cultures were used to inoculate 3 ml of LB broth kanamycin in round bottom 24-well plates and grown at 37 °C, 1050 rpm until OD600 reached ca. 0.8. Protein expression was induced with 0.5 mM IPTG and cultures were left overnight at 22 °C, 1050 rpm. Affimer proteins were purified from clarified lysates using His Mag Sepharose™ Ni beads on a KingFisher Flex™ robotic platform, as for SH2-domains with the exclusion of TCEP from the elution buffer. Samples were flash frozen in liquid nitrogen and stored in aliquots at − 80 °C.

### Microarray

Protein microarrays were conducted using HA-tagged Affimer reagents and BAP-tagged SH2 domain proteins. SH2 domain protein samples were diluted to 70 µM in PBS containing 20% glycerol and 10 µl samples added to wells in a 384-well microarray plate (Genetix). Proteins were spotted onto the surface of streptavidin-coated 3D-functionalized glass slides (PolyAn), using an ArrayJet Marathon™ non-contact printer. The system buffer contained 47% glycerol, 0.06% Triton™ X-100 (Sigma-Aldrich),0.04% ProClin™ 200 (Sigma-Aldrich) in ddH2O. Each protein spot consisted of 100 ρl solution, with a typical spot size of 200 μm. Proteins were left to dry onto the surface overnight, in a controlled environment of 18–19 °C and 50–55% humidity (using the ArrayJet JetMosphere™ system). Slides were scanned at 532 nm using a GenePix® 4300 A scanner (Molecular Devices) to visualise and analyse the printed protein spots for any drying artefacts. Slides were incubated with Blocking Buffer 1 (0.1 M Tris-HCl; 50 mM ethanolamine; 0.05% Tween-20, pH 9.0; 140 µl/well) for 15 min at room temperature. Wells were washed x3 with PBST and blocked additionally with Blocking Buffer 2 (2X Casein Blocking Buffer (Sigma-Aldrich); 0.1 M Tris-HCl, pH 8.5; 140 µl/well) for 30 min at room temperature. Arrays were then incubated with 5 µg/ml Affimer in Blocking Buffer 2 (70 µl/well) for 1 h at room temperature, followed by 3x PBST washes. Bound Affimer was detected using an anti-HA-tag AlexaFluorTM 647 conjugated antibody (1:1000; Thermo Fisher 26183-A647 diluted in Blocking Buffer 2 (70 µl/well), for 1 h at room temperature in the dark. Negative control miniarrays were included on each slide; these controls were incubated with Blocking Buffer 2 and HA-tag antibody only. Slides were washed 3 times with PBST, once with PBS and finally with ddH2O before centrifugation at 200x*g* for 5 min to dry. Slides were scanned at 635 nm using a GenePix® 4300 A scanner to detect bound HA-tag antibody. Images were analysed using image analysis software GenePix® Pro 7, which automatically detected spots and identified proteins according to the print layout. The local background signal surrounding each spot was also read to enable background correction for each spot. Each miniarray was analysed separately, with the mean fluorescence at 635 nm after subtraction of background fluorescence (F635 – B635) calculated for each SH2 target from the five replicate spots. For analysis of Affimer binding specificities, the F365 – B635 calculated for each SH2 protein spot against that Affimer clone was averaged over three 50 experimental repeats. The Affimer was considered to be a positive hit if the signal for the intended target was ≥ 50x that of the signal for the buffer-only control spot. Cross-reactions to other targets were deemed significant if the signal totalled ≥ 10% of the intended target signal.

### Purified protein ELISA

Purified protein ELISA were performed to test binding of HA-tagged Affimer proteins to their BAP-tagged SH2 target. Wells of Nunc-Immuno™ Maxisorp™ F96 plates were incubated with 50 µl of 5 µg/ml streptavidin (Molecular Probes® Life Technologies) in PBS at 4 °C overnight. Plates were blocked with Blocking Buffer overnight at 37 °C, washed with PBST, and 50 µl of 10 µg/ml SH2 protein in Blocking Buffer added per well. For streptavidin only controls, 50 µl of Blocking Buffer only was added. SH2s were incubated in the wells for 2 h at room temperature, followed by 1 x wash with PBST and incubation with 50 µl of 10 µg/ml Affimer protein in Blocking Buffer, for 1 h at room temperature. Each Affimer was tested against both SH2- containing and streptavidin-only wells. Wells were washed with PBST and incubated with 50 µl HA-tag antibody (1:20,000 ,Abcam, ab119703) in Blocking Buffer, for 1 h at room temperature. After 1 x wash with PBST, wells were incubated with 50 µl anti-mouse-HRP antibody (1:10,000; Abcam, ab6789) in Blocking Buffer for 1 h at room temperature. Plates were washed x 6 with PBST and HRP was detected using SeramunBlau® fast TMB (Seramun Diagnostica GmbH). Absorbance at 620 nm was read after 3 min and 10 min, before the reaction was stopped with 1 M H_2_SO_4_ and the absorbance read again at 450 nm.

### Cell culture

U-2 OS, HEK293 and HeLa cell lines (ATCC) were maintained in DMEM supplemented with 10% fetal bovine serum and 100U/mL penicillin-streptomycin at 37 °C in 5% CO_2_. The identity of all cell lines was verified by STR and all cell lines were mycoplasma negative.

### Plasmid transfections

Affimer DNA was subcloned from pBSTG into pCMV6-tGFP (Origene) using the Affimer-GFP forward and reverse primers. For reverse transfection with 50ng of Affimer DNA using Lipofectamine 2000 (100nl; Invitrogen; HEK293 and U-2 OS cells) or 100ng of Affimer DNA using X-Treme Gene 9 (300nl; Roche; HeLa cells) in 20 uL Opti-MEM were incubated in 96 well Viewpoint plates (PerkinElmer) for 20 min. 80 uL of cell suspensions were then added (1 × 10^4^cells/well for HEK293 and U-2 OS cells, and 5 × 10^3^ cells/well for HeLa cells).

### pERK translocation assay

pERK nuclear translocation was assessed as previously described^29^. Briefly, cells transiently transfected with GFP-tagged Affimer were starved for 1 h in serum-free media and stimulated with 25 ng/ml EGF for 5 min. Cells were rinsed in DPBS and fixed in 4% paraformaldehyde (PFA) for 15 min. Cells were then permeabilised with ice-cold methanol for 10 min at − 20 °C and rinsed with PBS before blocking in 1% milk for 10 min prior to incubation with anti-pERK antibody (1:100; Cell Signalling Technology 4370) in 1% milk for 1 h at room temperature. Cells were washed 3 times in PBS and incubated with Alexa-Flour 568 (1:1000; Molecular Probes, Invitrogen) and Hoechst 33342 (1:1000; Molecular Probes, Invitrogen) in 1% milk for 1 h at room temperature. Cells were washed 3 times in PBS and stored at 4 °C until imaging. Plates were imaged using ImageXpress® PICO automated cell imaging system (Molecular Devices) and analysed using MetaXpress® High-Content Image Acquisition and Analysis software (Molecular Devices).

### Fluorescence anisotropy

Fluorescence anisotropy (FA) assays were performed on Grb2-SH2 Affimers. All Affimer and Grb2 SH2 samples were dialysed into 50 mM Tris, 100mM NaCl, pH7.4 prior to use. Assays were set up in 96 well plates and analysed using a Tecan Spark™ 10 M microplate reader. 20 µM Affimer solutions were set up in triplicate and sequentially diluted by a factor of 2 across 12 wells. A fluorescein isothiocyanate-labelled phosphopeptide (FYp; FITC-GABA-S-pY-V-N-V-Q) was added to these wells to a final concentration of 20 nM. Grb2 SH2 protein was added to wells to a final concentration of 0.25 µM and following a 5 min incubation the anisotropy measured in each well at 24 °C (Excitation filter at 485 ± 20 nm, Emission filter at 535 ± 25 nm). Polarisation values for each Affimer concentration were plotted using a logarithmic scale (log10) for the concentration values, and the resultant sigmoidal curve fitted using the logistic function on Origin 9.1 software. From this fit, half maximal inhibitory values (IC50) values were calculated automatically by Origin.

### Surface plasmon resonance

Full-length Grb2 protein was expressed from a pET28a vector using the same method as SH2 domain expression. The protein also contained an N-terminal His tag and no BAP tag. All proteins used in Surface Plasmon Resonance (SPR) were further purified using S.E.C, which also functioned as a method to separate the Grb2 monomer from the dimer. Only monomeric fractions were used in SPR. Grb2 was diluted to 5 µg/ml in 10 mM Sodium Acetate, pH 5.6 and immobilised onto Amine-coupling chips (sensor chip CM5, GE Healthcare). Affimer concentrations of 6.25 nM–400 nM in SPR running buffer (50 mM Tris, 100 mM NaCl, 0.01% Tween-20, pH 7.4)were flowed over the immobilised Grb2 at a flow rate of 80 µl/min for 1–3 min in succession and binding was measured. A 1 M NaCl wash was used for chip regeneration between measurements. Binding curves were fitted using BIAevaluation 3.2 software and *K*_D_ values calculated from these. An activated flow cell containing no Grb2 that had been capped using ethanolamine was used as the blank.

### Protein extraction, immunoprecipitation and immunoblotting

Protein extraction, immunoprecipitation and immunoblotting were as previously described^51^. Briefly, cells were washed with ice-cold PBS and lysed in Mammalian Lysis Buffer (50 mM Tris; 150 mM NaCl; 1% (v/v) Nonidet P-40 (Sigma); pH 7.4) supplemented with HALT protease inhibitor cocktail and phosphatase inhibitor 2 (SigmaAldrich), for 30 min on ice, followed by centrifugation at 10,000 x*g* for 10 min at 4 °C. Protein concentrations were measured by BCA assay, as per manufacturer’s instructions (ThermoFisher).

For immunoprecipitation mammalian cells lysates, clarified lysates of His-tagged Affimer proteins produced in BL21 StarTM (DE3) *E coli*., His-Tag Dynabeads (ThermoFisher) and the Kingfisher Flex (ThermoFisher) were utilised. Dynabeads were incubated with 80 µl clarified lysate in 1x blocking buffer (SigmaAldrich) in wash buffer (100 mM Sodium-phosphate, pH 8.0, 600 mM NaCl, 0.02% Tween-20) for 10 min, and rinsed with wash buffer. Beads were then incubated with 500 µg mammalian cell lysate for 90 min at room temperature. Following three washes, proteins were eluted by incubation in His elution buffer (300 mM Imidazole, 50 mM Sodium phosphate, pH 8.0, 300 mM NaCl, 0.01% Tween-20) for 10 min. Immunoprecipitants were heated in 4xSDS-PAGE Sample Buffer (8% (w/v) SDS; 0.2 M Tris-HCl (pH 7); 20% glycerol; 1% bromophenol blue; 20% β-mercaptoethanol) and run on a 15% SDS-PAGE gels before transfer to nitrocellulose membrane using the BioRad Transblot Turbo. Membranes were then blocked in 5% milk in TBS-T before overnight incubation at 4 °C with rabbit Grb2 (1:5000, Abcam ab32037), or rabbit anti-6xHisTag-HRP (1:10,000 for 1 h at room temperature, Abcam, ab1187). Membranes were rinsed three times with TBS-T before 1 h incubation at room temperature with goat-anti-rabbit HRP (Abcam, ab97051) if required, followed by three more TBS-T rinses, and development using Immunoblot Forte Western HRP (Millipore), according to the manufacturer’s instructions. Blots were imaged using an Amersham™ Imager 600 (GE Healthcare, Chicago, IL). If required membranes were stripped with stripping buffer (0.2 M Glycine, 0.1% SDS, 1% Tween 20 (v/v) pH2.2) and rinsed three times with TBS-T before blocking and reprobing.

### Statistical analysis

Statistical analyses were carried out in GraphPad Prism 8.00 software (GraphPad Software, La Jolla, CA), with robust Z scores calculated in Microsoft Excel (Redmond, WA) as per the formulae in Birmingham et al.^52^. Statistical assumptions of equal variance for one-way ANOVA were tested with Brown-Forsythe tests. Fluorescent anisotropy data was plotted in Origin 9.1 software (OriginLab Corporation, Northampton, MA) and curves fitted with the logistical function.

## Electronic supplementary material

Below is the link to the electronic supplementary material.

Supplementary Material 1

## Data Availability

The datasets used and/or analyzed during the current study available from the corresponding author on reasonable request.
