# Supplementary material for: Generating and validating renewable affimer protein binding reagents targeting SH2 domains
Source: Sci Rep. 2024 Nov 16;14:28322. doi: 10.1038/s41598-024-79357-4 (PMC11569188; doi:10.1038/s41598-024-79357-4)
Supplement: Supplementary file 1 — Supplementary Material 1 [file 41598_2024_79357_MOESM1_ESM.docx]

**Supplementary Data**

**Supplementary Figure S1.**

**Variable Region 2**

**
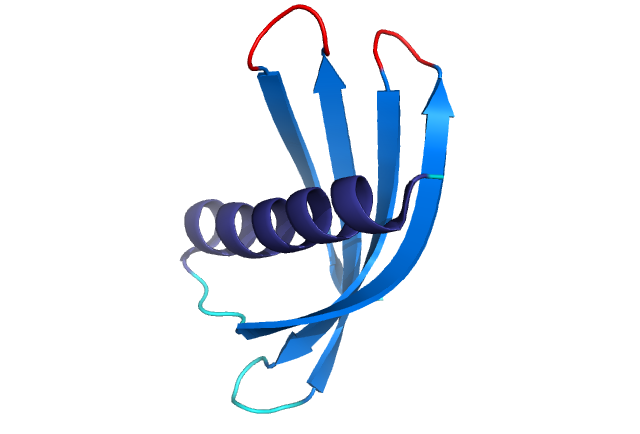
**

**Variable Region 1**

**Crystal structure of the Affimer scaffold shown as cartoon representation (PDB ID: 4N6T).** The α-helices and β-sheets are coloured blue, whilst the variable loop regions (VR) are shown in red (Tiede et al. 2014). Image was produced using PyMOL 2.0.

**
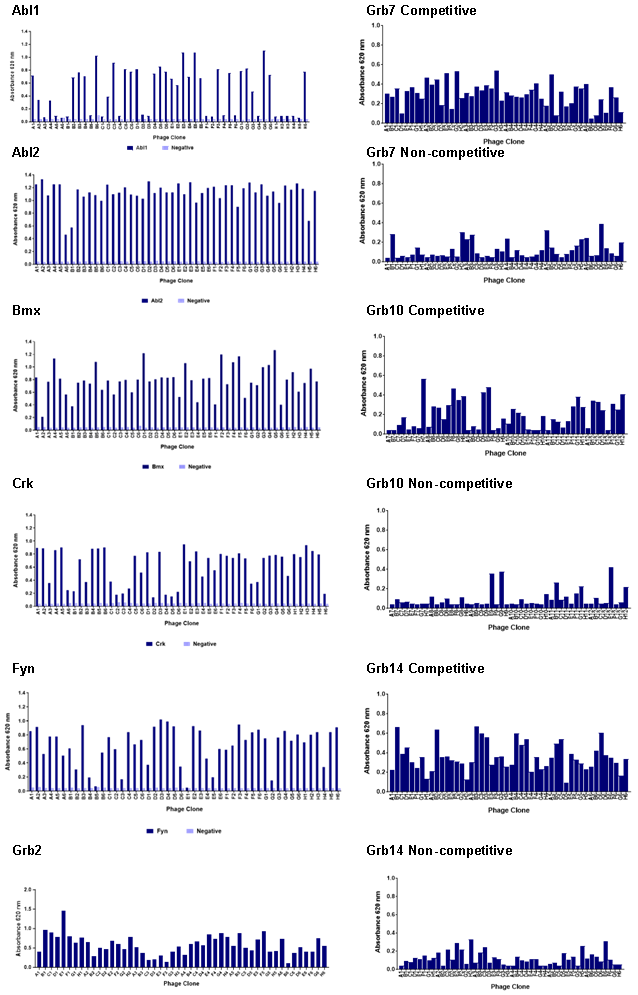
Supplementary Figure S2**

**
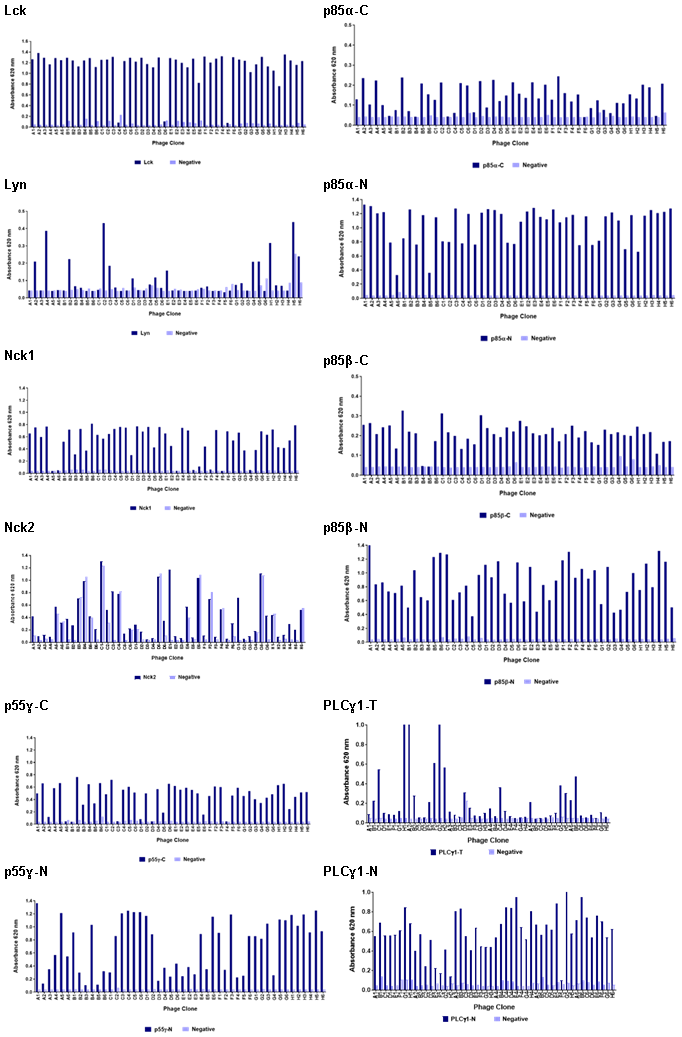

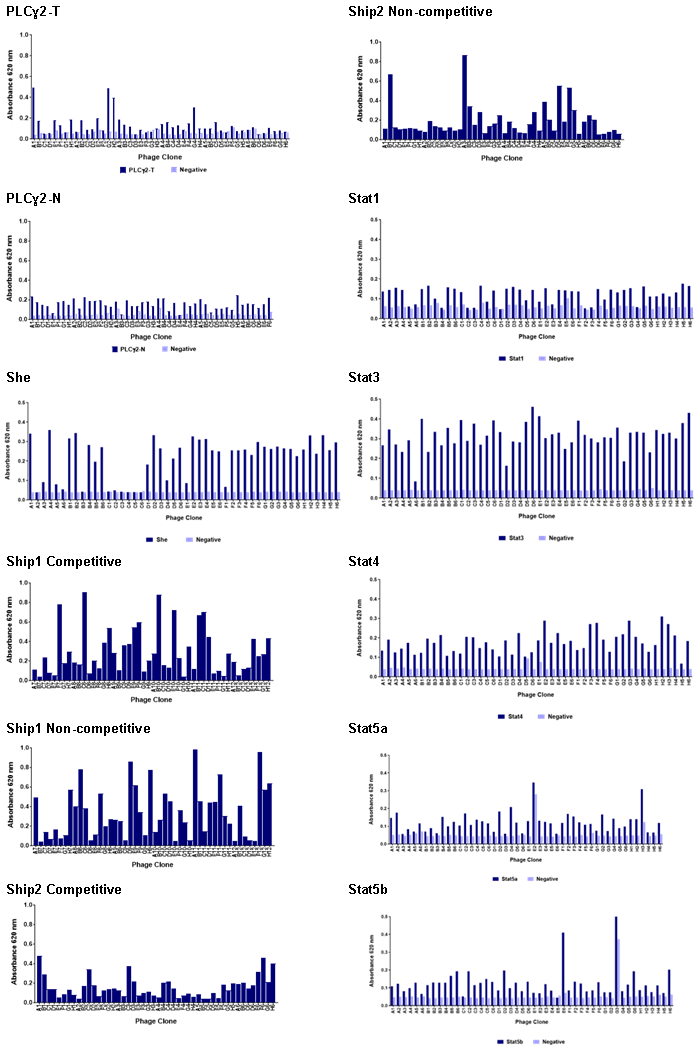

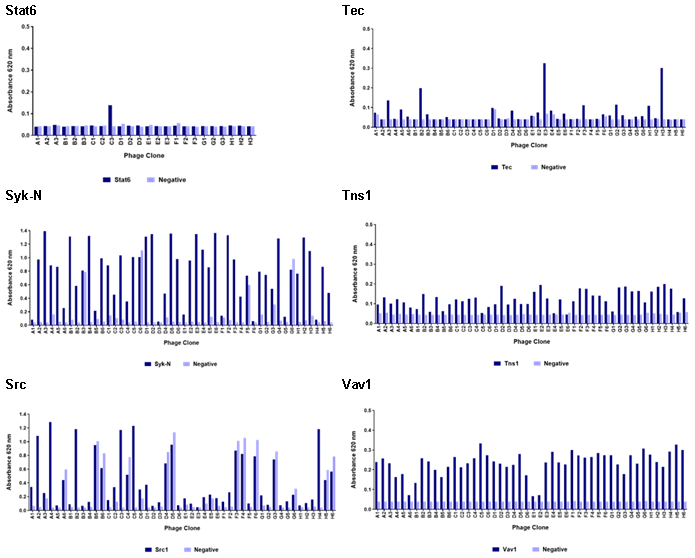
**

**Phage ELISA results from SH2 Affimer library screens.** Phage clones were incubated in wells containing immobilised SH2 and bound phage were detected with anti-phage-HRP antibody after washing. HRP substrate TMB was added and absorbance read at 620 nm after 3 min. 24 – 48 clones were tested for all SH2 domains. All screens were competitive, but for some targets a second non-competitive screen was also undertaken.

**Supplementary Figure S3
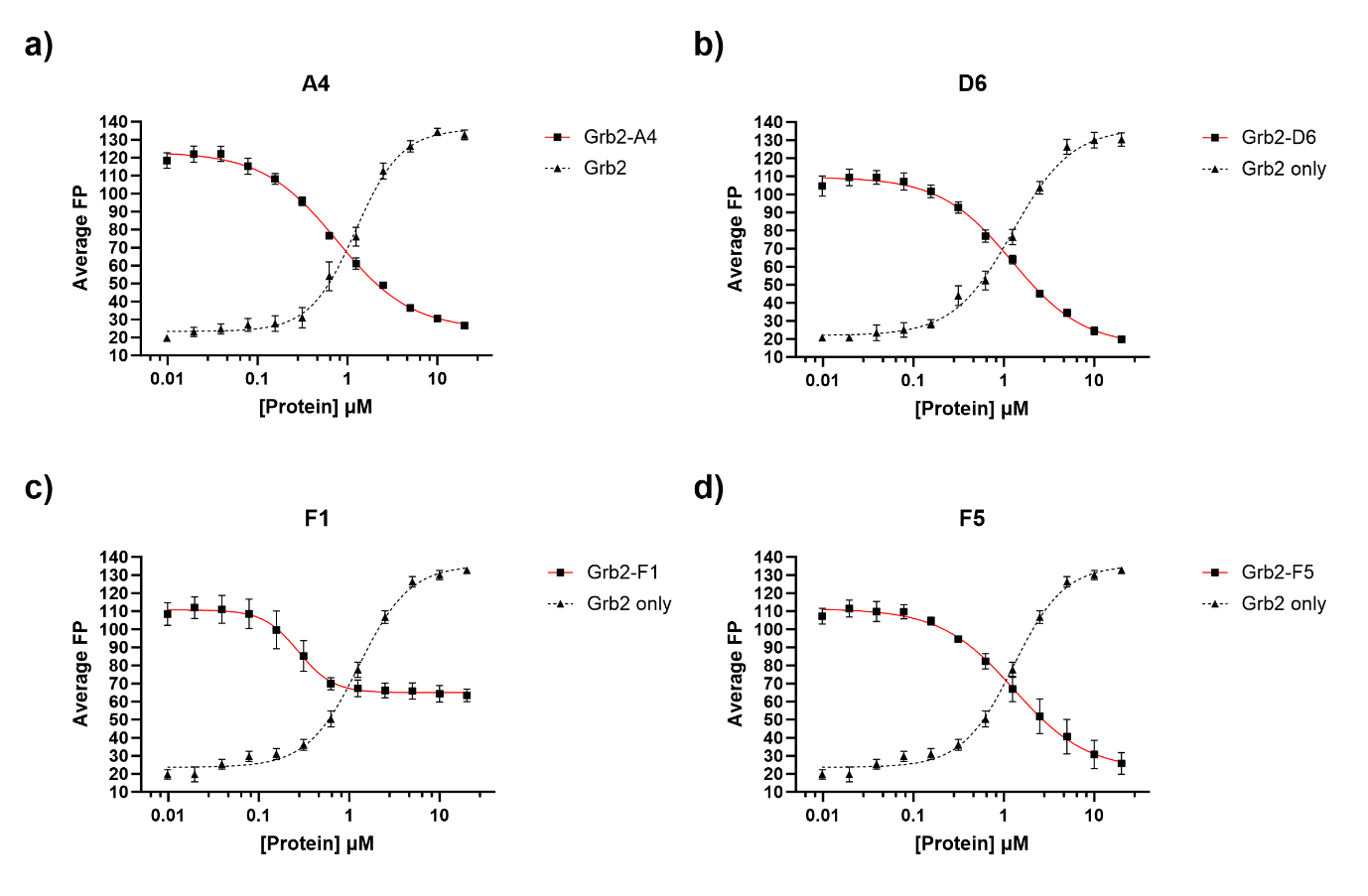
**

**Grb2 SH2-binding Affimers show competitive inhibition of the Grb2 SH2.** Fluorescence polarisation (FP) was used as a measure of binding between the Grb2 SH2 and a FITC-labelled phosphopeptide ligand (FYp). Serial dilutions of Affimer (A4 **a)**, D6 **b)**, F1 **c)** and F6 **d)**) were set up in triplicate and the FP measured in each well ([FYp] = 20nM; [Grb2 SH2] = 0.25µM). A Grb2 SH2-FYp control binding curve was also read on each plate ([FYp] = 20nM) with a serial dilution of Grb2 SH2 domain to ensure Grb2 SH2 and the FYp probe were binding as expected. Data shown is mean ± SEM. n = 3 independent experiments.

**Supplementary Figure S4**

**F1**

**a)** **b)**

**A4**

**Grb2 SH2-binding Affimer reagents show high binding affinity for their target**. Example SPR traces for Grb2 SH2 Affimer clones A4 (a)) and F1 (b)). Traces shown in grey for 100 nM Affimer bound to full-length immobilised Grb2. Binding curves were fitted using the 1:1 Langmuir binding model (shown in black). Injection time varied from 60 – 180 seconds for different clones. All graphs show 240 seconds dissociation phase. K_D_ values were calculated using a concentration range of 6.25 – 400 nM Affimer and show mean ± SD.

**Supplementary Figure S5.**


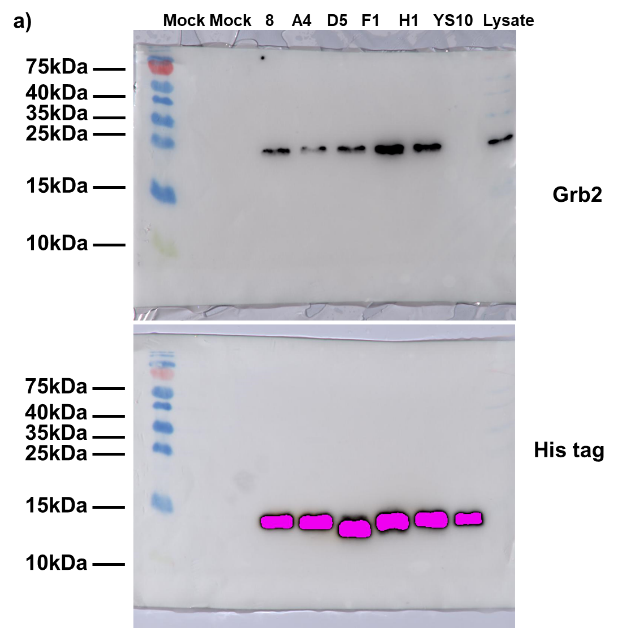


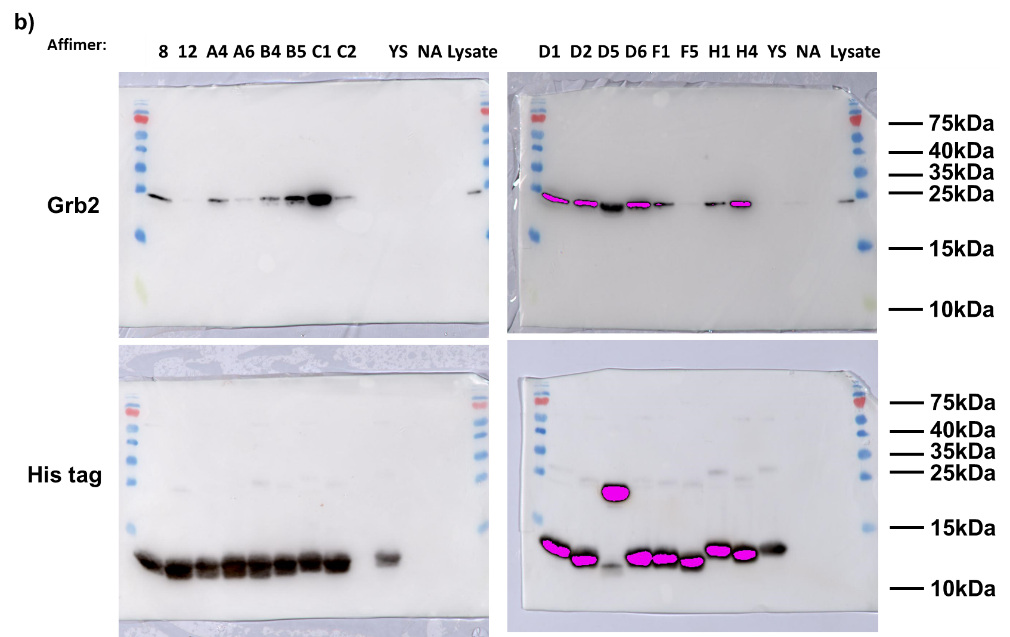


**c)**


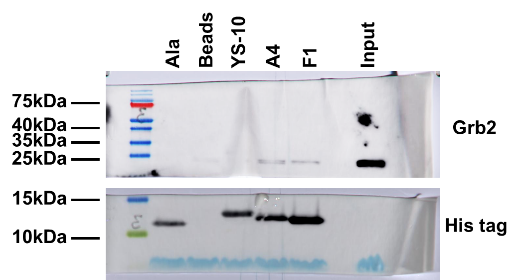


**Grb2 SH2-binding Affimer immunoprecipitation of Grb2.** Blots from which the representative lanes shown in Figure 3b (**a)**) and Figure 4a (**b)** and **c)**) for Grb2 Affimers A4 and F1, together with controls (YS/YS10 is a Affimer that binds yeast sumo; Ala is an Affimer with Variable region s of AAAA and AAE) were taken, showing the ability of the Grb2 SH2-binding Affimers to immunoprecipate Grb2 from cell lysates. Membranes were probed with anti-Grb2 before stripping and reprobing (see methods for details), except for blots in **c)** where the membrane was cut before probing. NA – no Affimer, beads only, YS – Yeast Sumo Affimer, Ala – Alanine Affimer.

**Supplementary Table S1. Target-specific Affimer clones as determined by SH2 protein microarray.** Table summarising specific Affimer clones for SH2 targets and their variable region sequences as identified by protein microarray (Figure 1). Clones were deemed specific if off-target interactions showed a signal <10% of that of the intended target. Residues shown in bold for Grb2 Affimers resemble the natural Grb2 binding motif of pYXNX.

| **SH2 Target** | **Affimer Clones** | **Variable Region 1** | **Variable Region 2** |
| --- | --- | --- | --- |
| Abl1 | C2 | NVVYVDAGF | EDEHIAIWF |
| Abl2 | E1 | QPLEWLELP | AAE |
|  | B3 | MPLDWLPMP | AAE |
|  | D4 | PPLPWLKVP | AAE |
|  | A2 | KALYYWPPD | NMGPDPMHH |
|  | A1 | APLDWLDLP | EDHNAGNFS |
| Bmx | G1 | IRYSSFATQ | RSMPMIKLH |
|  | A1 | FKYFSSHKI | RYQSIIHLK |
|  | D1 | GNIVQQWYH | DTPGMWHWN |
|  | A4 | YHEYQNGAF | WYPYNLWLK |
| Crk | A1 | FYDWPGNEYQSI | VAWMKNNVN |
|  | F3 | YDMPYPTVG | LWLKQYKGM |
|  | H3 | DWWNFPVFN | AAE |
| Fyn | D2 | QYLNSYWHG | KIMIEEDVY |
|  | A2 | DQKMDEYQD | YIFFDPWWV |
|  | D3 | YRNQSGDQD | YIYFSPWWV |
|  | A4 | IGEFAQKWA | EVWMDPWKV |
| Grb2 | A4 | HVL**WENA**GP | HTRYEYFVY |
|  | D6 | PW**YQNV**PYP | REERNMNAM |
|  | F1 | RW**YVNV**SLP | DNMDNMNKI |
|  | F5 | DWWEAGVFM | WNEINYMFD |
|  | H1 | RKL**WENY**KE | AMRMYYPEW |
| Grb10 | D9 (competitive) | SVTQPTRLQ | QWYFPMATM |
| Grb14 | F3 (competitive) | ARPEEPHWW | YKDNVYYFL |
|  | C3 (competitive) | EPKLYENQQ | PMVIPARWT |
| Lyn | A2 | AEEFMTFMG | QFLMPRMNL |
|  | C2 | PEGSGITVA | RWNMPKRFV |
|  | B2 | PEMMNVFWV | TYIMPPGRI |
| P85α-N | H2 | FEFNYNGQF | WIMLFDDGD |
|  | H1 (old screen) | WFTEVGPDH | QWLLPIHLM |
| P85β-N | E6 | AAVVAYITS | QEHWQHYMI |
| P55γ-C | C1 | LESQETVEF | LPQRLMTIW |
|  | B5 | PPGRAGIEW | LPHYLLTIW |
|  | G1 | PKYGEVSPH | IPHLILRIW |
|  | E5 | NMMHARRQW | EPHRLFVVW |
|  | E3 | YNSVDPHYD | MPHRLLTIW |
| P55γ-N | F2 | WTDRGPYDH | PFMNPLNLL |
| PLCγ1-N | B7 | WMDNFWRRM | QVHGNPWMD |
| PLCγ2-T | A1 | HRWWYDNFV | LAGHYAPSV |
| PLCγ2-N | A8 | HTFTWKWWY | NEDIESYEL |
| Ship1 | C8 (standard) | AHGPPDYHM | SIYFPMNYW |
| Ship2 | G5 (standard) | KYHDGYGPEPE | GLWWTPAHF |
| Stat3 | H2 | HGPVRVPWQ | DYGANLPLL |
|  | H6 | SPEEETPWA | SFQVNLQWI |
|  | B1 | EHDPTNPWT | RIQFHQQWH |
| Stat4 | F3 | FQNMWHSHS | QDLMLYQAP |
|  | H3 | TIHFRTFNS | EYIGNVFPM |
|  | H2 | HTFDYPAIH | PRIGKPKPK |
| Stat6 | C3 | HPLEMYEDE | VIYAWGGLM |
| Syk-N | D2 | NWQPLLSYW | PKTGAQELY |
|  | F2 | RTYPPFVFY | KNQNIFALY |

**Supplementary Table 2.** Primers used in this study.

| **Primer name** | **Primer sequence 5’ – 3’** |
| --- | --- |
| Affimer from pDHis to pET11 F | ATGGCTAGCAACTCCCTGGAAATCGAAG |
| Affimer from pDHis to pET11 R | TACCCTAGTGGTGATGATGGTGATGC |
| Affimer from pDHis to pCMV6-tGFP F | TATATGCGATCGCCATGGGTAACGAAAACTCCCTG |
| Affimer from pDHis to pCMV6-tGFP R | AATACGCGTAGCGTCACCAACCGGTTTG |
| Affimer (T1) from pDHis to pET-lectra F | TACACGTACTTAGTCGCTGAAGCTCTTCTATGATCC  CGCGTGGCC |
| Affimer (T1) from pDHis to pET-lectra R | TAGGTACGAACTCGATTGACGGCTCTTCTACCGAAA  CCCGTCAGCTCGTC |
| Affimer (T2) from pDHis to pET-lectra F | TACACGTACTTAGTCGCTGAAGCTCTTCTATGAGCGC  CGCTACCG |
| Affimer (T2) from pDHis to pET-lectra R | TAGGTACGAACTCGATTGACGGCTCTTCTACCGTCAC  CAACCGGTTTGAACTC |
